# Experimental investigation of freeze injury temperatures in trees and their contributing factors based on electrical impedance spectroscopy

**DOI:** 10.3389/fpls.2024.1326038

**Published:** 2024-02-13

**Authors:** Xinyu Song, Tong Gao, Mengyao Ai, Shan Gao

**Affiliations:** ^1^ College of Mechanical and Electrical Engineering, Northeast Forestry University, Harbin, China; ^2^ School of Civil Engineering and Transportation, Northeast Forestry University, Harbin, China

**Keywords:** freeze injury, semi-lethal temperature, electrical impedance spectroscopy, cooling rate, freezing duration

## Abstract

In trees, injuries resulting from subfreezing temperatures can cause damage to the cellular biofilm system, metabolic functions, and fibrous reticulum, and even cell death. Investigating the occurrence of freezing damage and its contributing factors could help understand the mechanisms underlying freezing injury and prevent the subsequent damage in trees. To achieve this, a laboratory experiment was conducted using cut wood samples from Korean pine (*Pinus koraiensis* Siebold & Zucc) and Simon poplar (*Populus simonii* Carr.), and the effects of environmental freezing factors, including freezing temperatures, freezing duration, and cooling rate, on the temperature at which freezing injuries occur were examined using the electrical impedance spectroscopy (EIS) method. The semi-lethal temperature (LT50), as an indicator of freezing injury in wood tissue, was theoretically deduced based on the measured extracellular resistance (*r*_e_) using EIS. The contributory factors to changes in LT50 were determined and their relationship was established. The results revealed that all freezing factors exhibited significant effects on electrical impedance characteristics (*r*_e_, *r*_i_, and τ), significantly influencing the LT50 of the wood. Random forest (RF) and support vector machine (SVM) models were used to assess the contribution of the freezing factors and moisture content (MC). Among the factors examined, freezing duration had the greatest impact on LT50, followed by the MC, whereas the contribution of the cooling rate was minimal. The model accuracies were 0.89 and 0.86 for Korean pine and Simon poplar, respectively. The findings of our study illustrate that the occurrence of freezing injury in trees is primarily influenced by the duration of freezing at specific subzero temperatures. Slow cooling combined with prolonged freezing at low subzero temperatures leads to earlier and more severe freezing damage.

## 1 Introduction

Low temperature is one of the most detrimental environmental stressors for trees in temperate or boreal zones. Freezing damage to wood tissue, resulting from chilling and freezing, has garnered significant attention owing to its significant financial impact on global wood utilization annually (Snyder and de Melo-Abreu 2005; Gale and Moyer, 2017; León-Chan et al., 2017; Ouyang et al., 2019a; Guan et al., 2023). Further, low temperature is a major abiotic stress factor with profound effects on plant growth and development (Ritonga and Chen, 2020; Rahimi-Ajdadi, 2022). Although temperate or boreal trees can tolerate cold stress during winter and early spring, extremely low subzero temperatures (−50°) or sudden and rapid temperature drops can cause freezing-induced injuries, hindering the growth of trees and wood productivity (Zhang et al., 2022). Freezing of wood tissues can lead to frost cracking and freezing damage, and even threaten the survival of trees (Pearce, 2001; Guy, 2003). The damage to wood cell membranes is attributed to ice formation and cellular dehydration resulting from chilling and freezing (Shammi et al., 2022; Tian et al., 2022). Deep supercooling or extracellular freezing is the mechanism whereby wood tissues and organs adapt to subfreezing temperatures to resist freezing damage (Gusta and Wisniewski, 2013; Wisniewski et al., 2014a). However, if the temperature continues to decrease, the deeply supercooled water will ultimately freeze, leading to lethal injury to the tissue (Ashworth and Davis, 1984).

When trees are exposed to subfreezing temperatures for extended periods of time, ice can form in the apoplast of wood tissues, causing water to move from the protoplasm to the ectoplasmic space (Verhoeven et al., 2018; Ramirez and Poppenberger, 2020). Prolonged exposure can result in a continuous increase in ice crystal volume, puncturing the cell membrane and potentially leading to cell death (Romero Fogué et al., 2022; Mihailova et al., 2020; Prerostova et al., 2021). In addition to the duration of exposure to freezing temperatures, the rate at which the environmental temperature decreases is another factor that affects ice formation in wood tissues (Weiser, 1970). When the cooling rate is rapid, the water in the protoplasm does not have sufficient time to exfiltrate, which can potentially lead to the formation of intracellular ice crystals. This results in freezing injury reactions, such as cell membrane disintegration as well as plasma outflow, causing further damage and potentially leading to cell death (Hofmann and Bruelheide, 2015; Shin et al., 2015).

Freezing injury in plants refers to the damage caused to the cellular biofilm system, metabolic functions, and fibrous reticulum due to mechanical stress and secondary drought, ultimately resulting in cell death. The semi-lethal temperature at which 50% of plant tissue is damaged or killed (LT50) is considered a critical indicator of freezing injury to woody tissue (Repo et al., 1997; Repo et al., 2021). The degree of freezing injury is influenced by factors such as the duration of exposure to freezing temperatures, intensity of chilling stress, rate of cooling, and location of ice formation (Lim et al., 1998; Beck et al., 2004; Fujikawa et al., 2018; Kovaleski and Grossman, 2021). Therefore, understanding structural changes that occur in wood tissues at subfreezing temperatures is of practical importance for investigating the mechanism underlying freezing injury in trees.

Various methods have been used to assess freeze-injury in plant branches, shoots, and seedlings during controlled laboratory experiments (Endoh et al., 2014; Palacio et al., 2015). Freezing of water is an exothermic process. Differential thermal scanning (DSC) analysis has been used to determine the freezing temperature in wood tissues; previous studies have determined freezing time by observing the region of exothermic reactions in DSC curves (Neuner et al., 2010; Arias et al., 2015; Arias et al., 2017). Freezing processes in wood tissues typically involve two exothermic reactions: the first occurs at relatively higher temperatures and involves freezing of water in the extracellular spaces (Repo et al., 2022), whereas the second occurs at considerably lower temperatures and involves the freezing of supercooled water in intracellular spaces. In the case of some boreal and cold-temperate tree species, intracellular water is susceptible to supercooling and deep supercooling. In these species, tissue cells demonstrate resistance to ice nucleation activity and freezing of supercooled water within these cells is associated with the occurrence of exothermic processes at considerably lower temperatures. Some tree species are likely to undergo multiple exothermic reactions at lower temperatures (Räisänen et al., 2006; Repo et al., 2022).

In recent years, electrical impedance spectroscopy (EIS) has emerged as a nondestructive detection method widely used to assess physiological changes in various wood cells, tissues, and whole plants in response to freezing stress, cold acclimation, and decay (Luoranen et al., 2004; Wu et al., 2008; Zhang et al., 2010; Yue et al., 2018). The electrical impedance of the cellular structure of tissues comprises resistance and capacitance; the electrical impedance exhibits variation across different frequency ranges, and the EIS profile and its parameters directly reflect the physiological changes occurring in tissue cells (Ando et al., 2014; Repo et al., 2016; Jócsák et al., 2019; Wu et al., 2019). EIS parameters have been used to evaluate plant cold resistance by converting them into temperature response inflection points (LT50) (Repo et al., 2000; Wu et al., 2008; Zhang et al., 2010; Repo et al., 2021).

The northeast forest area (118°–135°E, 48°–55°N) constitutes the largest natural forest area in China, covering approximately 37% of the nation’s total forested land. Trees in this region endure prolonged periods of subfreezing temperatures during the winter season. The historical lowest temperature recorded in this area reached a staggering −55.1°C. Freezing injury can occur in woody plants exposed to extreme low temperature.

Trees exposed to subfreezing temperatures for a long time can cause brittle breakage, frost cracking, large stuttering, and even death. Investigation of the temperature and conditions of freezing damage occurrence is the basis for understanding the mechanism underlying freezing damage in trees, which is of great value for the resistance and acclimation of precious tree species to cold conditions. In the present study, we used EIS to evaluate the LT50 and cytoarchitectural states during freezing in the sapwood tissues of trees. We examined the effects of freezing factors on EIS characteristics and the LT50 of wood.

The objectives of this study were: (1) to investigate the critical temperature at which the freeze injury occurs in the trees subjected to subfreezing temperature environments by modelling the relationship between EIS characteristics and LT50, and (2) to examine the effects of exposure to subzero temperatures, freezing duration, cooling rate, and moisture on the LT50 of sapwood tissue.

## 2 Materials and methods

### 2.1 Materials and sample preparation

**Figure 1:**
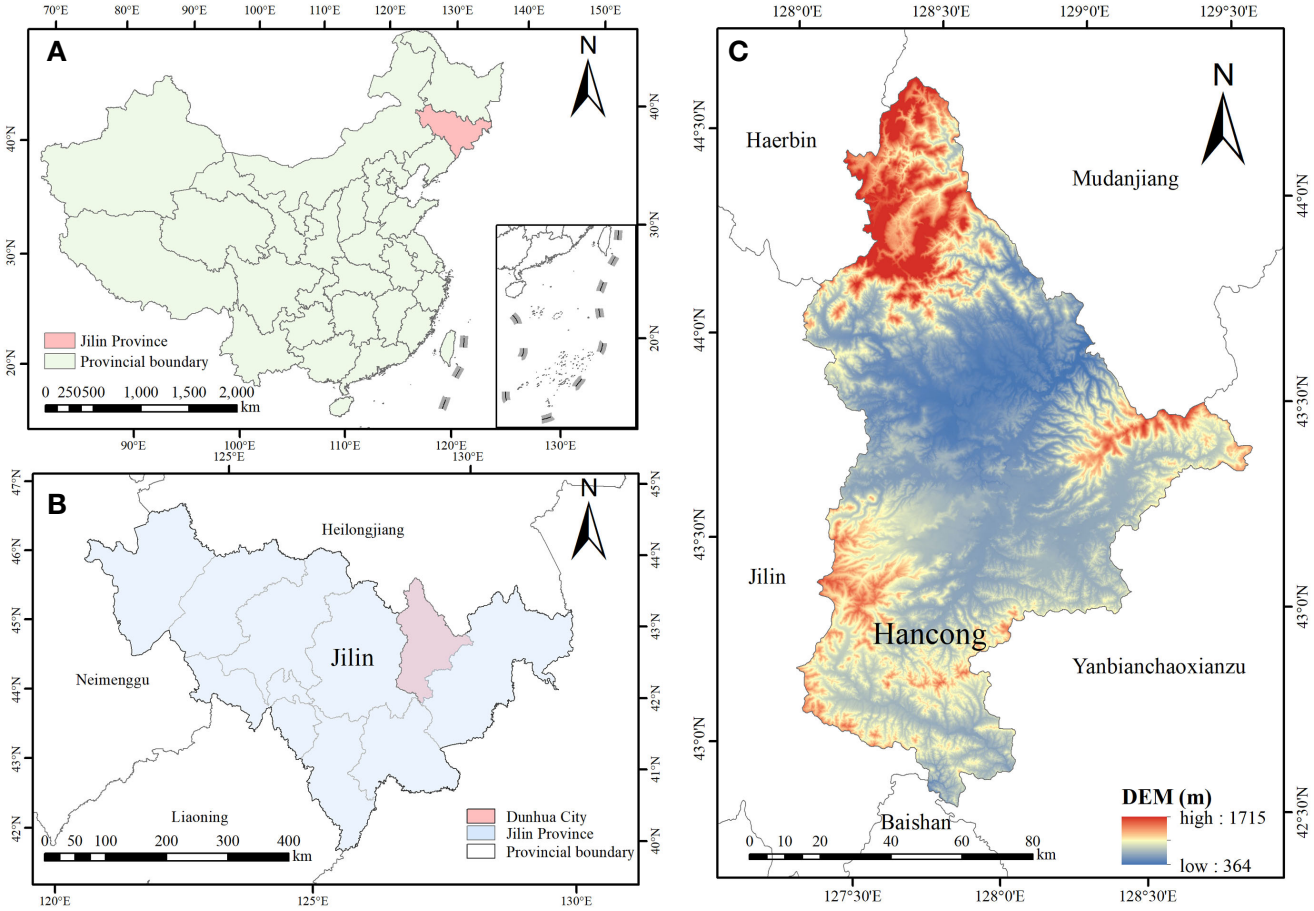
Geographical location maps of the Hancong forest region in Changbai Mountains. **(A)** The location of Changbai Moutains in China. **(B)** The location of Hancong Ridge in Jilin Province. **(C)** Digital elevation model (DEM) of Hancong Ridge.

**Figure 2:**
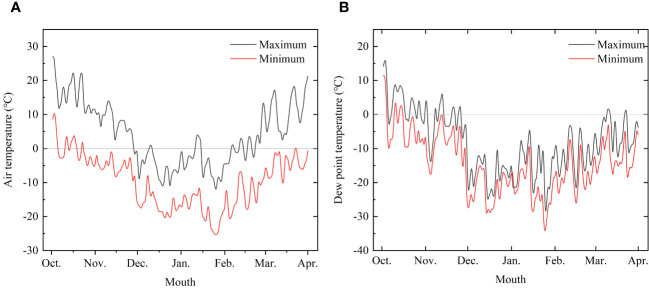
Temperature change trend monitored from October 2022 to March 2023. **(A)** Air temperature and **(B)** Dew point temperature.

The experiments were conducted at the laboratory and logs of Korean pine (*Pinus koraiensis* Siebold & Zucc) and Simon poplar (*Populus simonii* Carr.) species were used for the experiments. The logs were obtained from the Hancong Ridge Forests in the Changbai Mountains, China. The Hancong Ridge Forests are located in the northeast region of China (**Figure 1**), and the area experiences subfreezing temperatures for over four months of the year (**Figure 2**). The logs used in this study were cut from standing trees on-site with a diameter of approximately 25 cm. They were then processed into small, flawless cubes of wood measuring 10 × 10 × 10 mm^3^, separated into heartwood and sapwood. The cubes were numbered and wrapped in plastic film for preservation until further analysis.

### 2.2 Moisture content conditions

To determine the effect of the MC, we conditioned the wood samples to six MC levels including air-dry (15%), fiber saturation point (30%), and fully saturated wood (80%, 100%, 150% and 200%). Each group corresponding to a specific MC level consisted of 60 wood samples. All samples were air-dried naturally in a ventilated, cool place of the laboratory. A sample was randomly selected from each group and oven-dried at 105°C for 24 hours. During the drying process, six samples were weighed every 2 h until the weight difference between two consecutive measurements did not exceed 0.0002 g. The weights of the six samples were considered as completely dried weights of the corresponding group and recorded as *M*_0_. The dried samples were stored in weighing bottles with a desiccant.

The target weight of each sample (*M*_i,_ *_i_*_=1,2,3…6_) at each MC level was calculated using the Equation 1:


(1) $$MC=\frac{M_{i}-M_{0}}{M_{0}}$$

Following this, all dried samples were soaked in beakers containing deionized water and sealed with Parafilm (Parafilm “M” Laboratory Film PM996, Bemis, WI, USA). After soaking for 12 h, the samples were air-dried with natural air. Five samples from each group were randomly weighed every 0.5 h and the weight was recorded until it approached the target weight *M*_i_ over time. Finally, the conditioned samples were individually wrapped with plastic film to prevent moisture loss.

### 2.3 Differential scanning calorimetry measurements

The selected samples from the six MC groups were cut with a sharp blade into smaller cubic shapes measuring 2 × 2 × 2 mm^3^. Two cooling rates, namely a slow rate of 2°C/min and a fast rate of 10°C/min, were set for the DSC tests. The samples were grouped based on the species and cooling rate into four groups: *Pinus*–slow-freezing (PiS), *Pinus*–fast-freezing (PiF), *Populus*–slow-freezing (PoS), and *Populus*–fast-freezing (PoF). To prepare the samples for the DSC, the samples were weighed and placed in a Φ5.4 mm × 2.6 mm crucible (TA-JYL0010, TA instruments, DE, USA). The high temperature exotherms and low temperature exotherms (HTEs and LTEs) during freezing were measured using the DSC device (TA Instruments Q20, TA instruments, DE, USA), starting from an initial temperature of 10°C. Each group of samples was cooled down to −80°C at both cooling rates, with eight repetitions at each rate. The obtained DSC curves were analyzed to determine the onset and termination temperatures of HTEs and LTEs, as well as the corresponding exothermic peaks.

### 2.4 Freezing condition control

**Figure 3:**
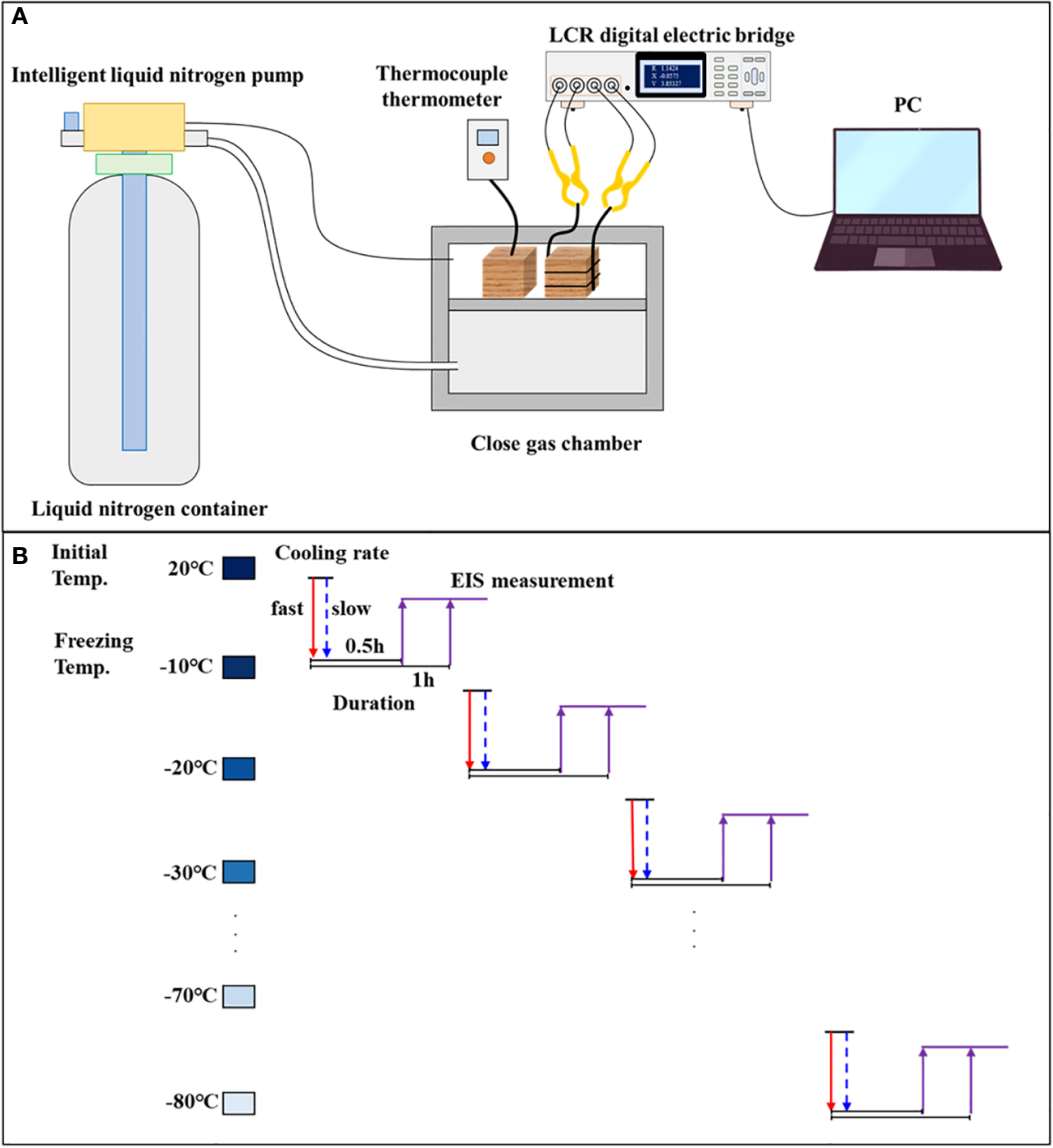
**(A)** Schematic diagram of the freezing system and **(B)** the flowchart of EIS measurement.

A gas chamber was built as a sealed freezing room for wood sample temperature control. Liquid N_2_ was injected into the gas chamber using a pump, and the flow rate of liquid N_2_ was controlled by adjusting the pump pressure (**Figure 3A**). To align with the conditions set for the DSC tests, the cooling rates of 2°C/min and 10°C/min were maintained. Each species–cooling rate group (PiF, PiS, PoF, and PoS) comprised 48 samples. The samples were conditioned to the target MC level and then cooled down in an orderly manner to −10, −20, −30, −40, −50, −60, −70, and −80°C at both slow and fast cooling rates (**Figure 3B**). Thermocouples were connected to the samples to monitor the temperature changes during freezing, and liquid N2 was automatically refilled to maintain the desired temperature and cooling rates. The freezing duration for each sample at every temperature point was 0.5 and 1 h.

### 2.5 Electrical impedance spectroscopy

Conductive paint was evenly applied to both radial facets of the sample section. One end of a conductive wire was fixed on the surface of the wood sample using insulating rubber and the other end was attached to insulating jigs (TH26011BS, Tonghui Electronic Co. Ltd., Changzhou, China) using tinned copper. The impedance spectrum, including the real part (*ZRe*) and the imaginary part (*ZIm*), was measured using a high-frequency LCR digital bridge (Tong-Hui TH2826/A, Tonghui Electronic Co. Ltd., Changzhou, China) in the frequency range of 80 Hz to 1 MHz (**Figure 3**). The input voltage of the sine signal was set to 100 mV. The EIS parameters were modeled using the single-DCE model in the distributed circuit model based on *ZRe* and *ZIm*. The model is described by Equation 2 as follows:


(2) $$Z=R_{\infty }+\frac{R}{1+{\left(i\tau \omega \right)}^{\psi }},$$

where *R*_∞_ (Ω) represents the resistance at high frequencies, *R* (Ω) is the difference between the direct current resistance and *R*_∞_ (Ω), *i* is the imaginary unit, ω (*2πf*) is the angular velocity, τ is the relaxation time, and ψ is the distribution coefficient.

The model parameters were fitted using ZSimpWin 3.60 software (ZSimpWin, AMETEK, Inc., USA). At low frequencies, the electrical double layer prevents the current from passing through the cell membrane, and it flows only in the apoplastic space (Ehosioke et al., 2020). The extracellular resistance (*R*_e_) was calculated using Equation 3:


(3) $$R_{e}=R_{\infty }+R$$

At high frequencies, the currents can pass through the cell membrane, flowing in both the apoplastic and symplastic spaces (Ehosioke et al., 2020). The intracellular resistance (*R*_i_) is given by Equation 4:


(4) $$R_{I}=R_{\infty }\left(1+\frac{R_{\infty }}{R}\right)$$

The resistance parameter (*R_e_*_/_ *_i_*) was normalized to the cross-sectional area (*A*, m^2^) and length (*l*, m) of the sample to obtain the corresponding specific resistance value (*r_e_*_/_ *_i_*) using Equation 5:


(5) $$r_{e/i}=\frac{A}{l}\times R_{e/i}$$

### 2.6 LT50 estimation based on parameter *r*_e_

Specific extracellular resistance (*r*_e_) is a measure of the leaching of symplastic electrolytes to the apoplastic space as a result of cell membrane injuries (Repo et al., 2021). To estimate the LT50 value, the specific extracellular resistance (*r*_e_) was modeled using a logistic sigmoid function with respect to the exposure temperature, using the MATLABR2021 software (Equation 6).


(6) $$y=\frac{A}{{\left(1+e\right)}^{B(C-x)}}+D$$

In this equation, *y* represents parameter *r*_e_, and *x* corresponds to the exposure temperature. The parameters *A* and *D* define the asymptotes of the sigmoid curve, and parameter *B* represents the slope of the curve at inflection point *C*. The temperature corresponding to the inflection point *C* of the sigmoid curve obtained from Equation 6 is considered the LT50 value (Repo et al., 1994; Sutinen et al., 1992; Wu et al., 2019; Zhang et al., 2010). The initial values of *A*, *B*, *C*, and *D* in Equation 6 were fitted using the 1stopt5.0 software (First Optimization, 7D-Soft High Technology Inc., Beijing, China). The LT50 value (parameter *C* in Equation 6) of the wood sample was determined through measured electrical impedance spectroscopy using nonlinear regression analysis.

### 2.7 Statistical analysis

The interactive effects of temperature, MC, cooling rate, and freezing duration on EIS characteristics values and LT50 were statistically analyzed and evaluated using software SPSS (IBM, Armonk, NY, USA). A repeated measures one-way ANOVA was used to test significant differences in EIS characteristics (*r*_e_, *r_i_*, τ) and LT50 between MC, cooling rate, and freezing duration. The significance differences of LT50 between MC were compared using Duncan Multiple Range Test at a significance level of p< 0.05.

## 3 Results

### 3.1 Thermal behaviors and transitions of sapwood during the freezing process

**Figure 4:**
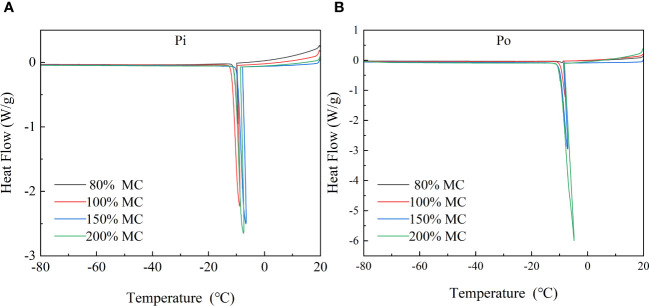
DSC curves of pine and poplar wood during controlled cooling at a rate of 2°C/min (from 20°C to −80°C). **(A)** DSC curves of pine and **(B)** DSC curves of poplar.

The DSC curves of wood samples during the freezing process are shown in **Figure 4**. HTEs occurred at a temperature of approximately −10°C for all wood samples of both species with MC over 70%. Samples with MC below fiber saturation point (FSP) exhibited rounded curves, indicating the transition of free water (extracellular) in the sapwood tissue from liquid to solid starting at −10°C. However, no LTEs were detected in any of the wood samples. This finding suggests that the DSC method is not suitable for detecting the transition of the intracellular water or changes in the membrane structure of wood cells.

### 3.2 EIS profiles during the freezing process

#### 3.2.1 Observation of extracellular resistance

**Figure 5:**
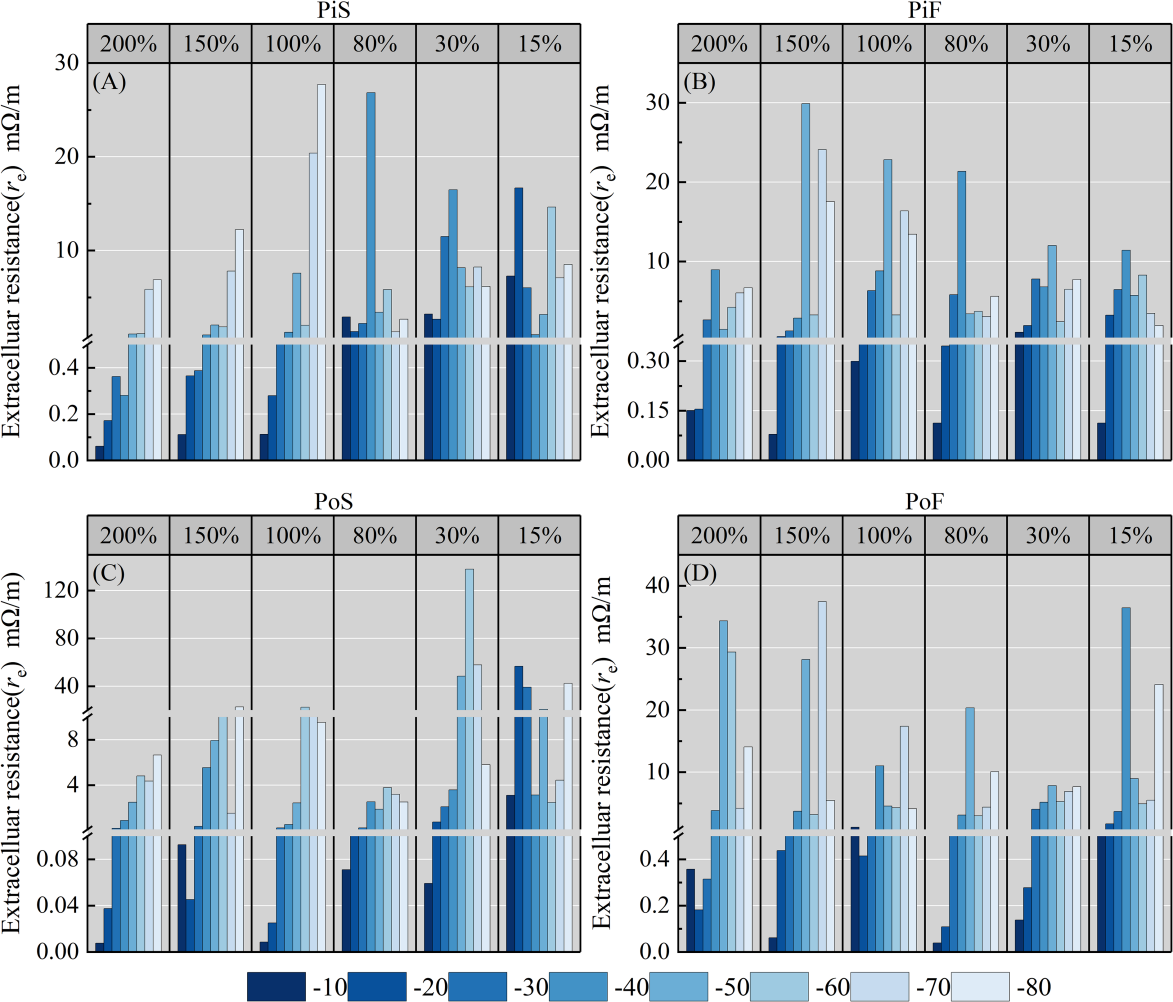
Variation in *r*_e_ with temperature for different moisture content levels and cooling rates during the freezing process. **(A)** pine–slow cooling rate, **(B)** poplar–slow cooling rate, **(C)** pine–fast cooling rate, and **(D)** poplar–fast cooling rate.

The extracellular resistance *r*_e_ of both species at different MC levels and temperature points is illustrated in **Figure 5**. It is evident that *r*_e_ exhibits an overall increasing trend at all subzero temperature points compared to −10°C, and the extent of increase varies with the MC. In the groups subjected to slow cooling rates, the maximum increase in *r*_e_ for pine (PiS group) (**Figure 5A**) mainly occurred at −40°C for 80% MC and at −70 to −80°C for MC over 100%. The *r*_e_ value for 30% and 10% MC were relatively smaller, with the maximum increase occurring at approximately −40°C. Poplar (PoS group) (**Figure 5C**) showed a trend similar to that observed for pine for all MC levels, with −40°C and −70 to -80°C serving as turning points for the impedance measured at slow cooling rates.

In the groups subjected to fast cooling rates, the maximum increase in *r*_e_ for pine (Pif group) (**Figure 5B**) mainly occurred at approximately −40°C for all MC levels, followed by temperatures of −70 to −80°C for MC over 80%. Poplar and pine (PoF group) (**Figure 5D**) showed similar trends to those observed for the slow cooling rate groups for all MC levels.

In the present study, the measured intracellular resistance (*r*_i_) was only 10^-6^ to 10^-7^ times that of the *r*_e_ and the transition temperature of *r*_i_ was not clearly observed for either species under all freezing conditions. Therefore, intracellular resistance was not deemed suitable for estimating the LT50.

#### 3.2.2 Observation of the relaxation time of wood

**Figure 6:**
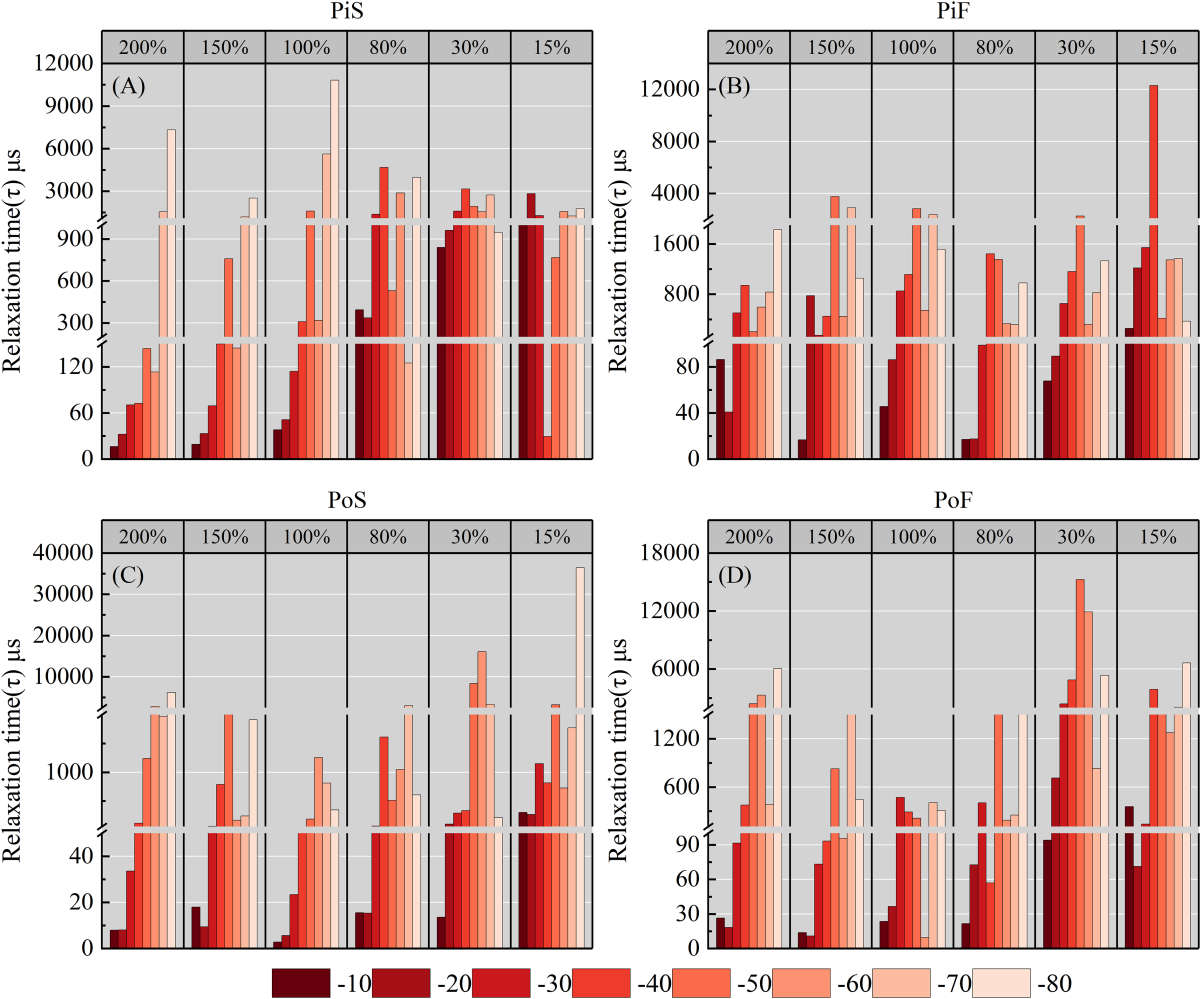
Variation of *τ* in sapwood tissues with different moisture content with changing temperatures. **(A)** pine–slow cooling rate, **(B)** poplar–slow cooling rate, **(C)** pine–fast cooling rate, and **(D)** poplar–fast cooling rate.

The relaxation time (*τ*) of both species generally increased as the temperature decreased for all MC levels at fast and slow cooling rates (**Figure 6**). The behavior of *τ* differed for pine with different MC levels in the PiS group. A greater increase in *τ* occurred around −70 to -80°C, followed by −40 to −50°C for slow cooling rates. In the PiF group, tissues with MC ≥ 30% showed significant changes in *τ* in the ranges of −40 to −50° and −60 to −70°. Tissues with MC of 10% showed a significant increase in *τ* only at −40°. These results indicate that the *r_e_* and *τ* varied significantly with changes in the temperature and MC.

The ANOVA results revealed that subzero temperature, MC, and cooling rate exhibited highly significant effects on *r*_e_, *r*_i_, and *τ* of the sapwood tissues (**Table 1**). Additionally, their interactions also had a significant effect on the *r*_e_, *r*_i_, and *τ* of the sapwood tissues.

**Table 1:** A repeated measures ANOVA of MC, freezing duration and cooling rate on EIS characteristics (*r*_e_, *r_i_*, τ).

Source of variation	Pi						Po					
	*r_e_*		*r_i_*		τ		*r_e_*		*r_i_*		τ	
	*F*	*P*	*F*	*P*	*F*	*P*	*F*	*P*	*F*	*P*	*F*	*P*
MC	1670.199	0.000**	98.549	0.000**	524.827	0.000**	335.501	0.000**	45.7390	0.000**	908.567	0.000**
T	4159.020	0.000**	98.507	0.000**	1895.976	0.000**	513.056	0.000**	16.035	0.000**	795.155	0.000**
FR	1355.403	0.000**	434.501	0.000**	634.760	0.000**	213.267	0.000**	116.688	0.000**	84.245	0.000**
MC/T	1614.349	0.000**	35.328	0.000**	647.100	0.000**	239.823	0.000**	10.685	0.000**	461.684	0.000**
MC/CR	2051.950	0.000**	74.786	0.000**	705.423	0.000**	195.802	0.000**	35.415	0.000**	206.478	0.000**
T/CR	1679.400	0.000**	94.150	0.000**	1185.106	0.000**	149.030	0.000**	8.771	0.000**	154.432	0.000**
MC/T/CR	1802.738	0.000**	56.226	0.000**	597.734	0.000**	224.383	0.000**	10.352	0.000**	207.052	0.000**

**P< 0.01.

*F*-values and *p*-values for source of variation in extracellular resistivity (*r*_e_), intracellular resistivity (*r*_i_) and relaxation time (*τ*) estimates of pine (Pi) and poplar (Po) sapwood tissues based on the moisture content (MC), temperature (T), cooling rate (CR), and their statistically significant interactions.

#### 3.2.3 Interactive effects of freezing factors and MC on LT50 based on *r_e_*

The corresponding LT50 values of sapwood at each transition temperature for both species were estimated based on the parameter *r*_e_ using Equation 6. The LT50 values at different MC levels and cooling rates were calculated and are presented in **Table 2**.

**Table 2:** One-way ANOVA of moisture content (MC) on LT50. LT50 values of sapwood tissues from pine (Pi) and poplar (Po) with different moisture contents after freezing at two rates for 0.5 h and 1 h.

TS	FR	MC	Duration		TS	FR	MC	Duration	
			0.5 h	1 h				0.5 h	1 h
Pi	Slow	200%	−60.50 ± 1.55a	−43.86 ± 2.91a	Po	Slow	200%	−57.23 ± 1.36a	−54.86 ± 1.34a
		150%	−57.31 ± 1.29b	−41.29 ± 2.27a			150%	−58.25 ± 1.59a	−54.34 ± 2.76ab
		100%	−52.83 ± 2.37c	−40.68 ± 2.03a			100%	−56.78 ± 3.10a	−51.27 ± 1.53b
		80%	−51.62 ± 1.67cd	−32.13 ± 2.34b			80%	−41.58 ± 1.50c	−36.49 ± 2.39d
		30%	−49.44 ± 1.52d	−25.13 ± 1.75c			30%	−50.98 ± 1.40b	−44.16 ± 1.41c
		10%	−50.30 ± 1.71cd	−23.88 ± 1.90c			10%	−40.81 ± 1.78c	−33.77 ± 1.66d
	Fast	200%	−41.02 ± 1.49ab	−30.35 ± 1.56a		Fast	200%	−47.61 ± 1.59a	−41.98 ± 1.49ab
		150%	−42.61 ± 1.24a	−31.20 ± 1.44a			150%	−49.93 ± 1.99a	−42.41 ± 1.95ab
		100%	−43.02 ± 1.76a	−31.82 ± 1.48a			100%	−48.02 ± 1.74a	−43.16 ± 0.91a
		80%	−39.09 ± 1.55b	−29.78 ± 1.90a			80%	−47.80 ± 1.32a	−39.81 ± 1.51b
		30%	−40.02 ± 1.99ab	−21.05 ± 1.93b			30%	−44.00 ± 1.94b	−32.14 ± 1.59c
		10%	−38.10 ± 1.28b	−20.00 ± 1.97b			10%	−44.36 ± 1.58b	−30.60 ± 1.52c

Different letters indicate significant differences between subspecies (*P*< 0.05).

For a freezing duration of 0.5 h, the LT50 values for pine at a slow cooling rate (PiS group) and poplar at a slow cooling rate (PoS group) ranged from −30 to −60°C, decreasing as the MC increased from 80% to 200% when the MC exceeded the FSP. The LT50 values for pine at a fast-cooling rate (PiF group) ranged from −30 to −40°C and for poplar at a fast growing rate (PoF group), they ranged from −30 to −50°C. The FSP did not behave as a distinct point for changes in LT50 change with different MC levels. For a freezing duration of 1 h, LT50 values for pine at both slow and fast cooling rates ranged from −20 to −40°C, decreasing with increasing MC levels. The LT50 values for poplar ranged from −30 to −50°C at a slow cooling rate and from −30 to −40°C at a fast rate. The LT50 values from pine wood samples frozen for 1 h were approximately 30% higher on average than those frozen for 0.5 h, with a maximum increase of approximately 53%. For poplar wood, the LT50 values for a 1-h freezing duration were 8% higher on average than those for a 0.5-h freezing duration, with a maximum increase of approximately 15.61% for 10% MC. The increased LT50 values with longer freezing duration indicates that the longer trees survive at certain subzero temperatures, the earlier the LT50 appears for wood tissue. The difference in LT50 between the MC levels in the PiS–0.5 group was minimal. Furthermore, the FSP did not act as a distinct point for changes in LT50 with different MC levels.

A comparison of LT50 values between the slow and fast cooling rate groups revealed that the LT50 values for both species at a slow cooling rate were slightly lower than those at a fast rate for freezing durations of both 0.5 h and 1 h, except the LT50 value for samples with 80% MC, which was slightly higher than that in the fast-cooling rate group.

The results revealed that the MC, freezing duration, and cooling rate had significant effects on the LT50 values of sapwood tissues (**Table 3**). The ANOVA results confirmed the significant influence of the MC, freezing duration, and cooling rate, and their interactions on the LT50 values of sapwood tissues (*p<* 0.0001) (**Table 3**).

**Table 3:** A repeated measures ANOVA of MC, freezing duration and cooling rate on LT50 for Pine and Poplar.

Source of variation	Pi		Po	
	*F*	*P*	*F*	*P*
Moisture Content (MC)	67.32	0.000**	126.22	0.000**
Duration (D)	1515.73	0.000**	269.72	0.000**
Cooing Rate (CR)	483.39	0.000**	187.70	0.000**
MC/D	27.77	0.000**	5.91	0.000**
MC/CR	16.78	0.000**	41.37	0.000**
D/CR	30.07	0.000**	17.31	0.000**
MC/D/CR	2.57	0.038	1.44	0.224

**P< 0.01.

*F*-values and *p*-values for the LT50 estimates for MC, freezing duration, cooling rate, and their statistically significant interactions.

#### 3.2.4 Prediction of LT50 based on the MC and freezing factors

**Figure 7:**
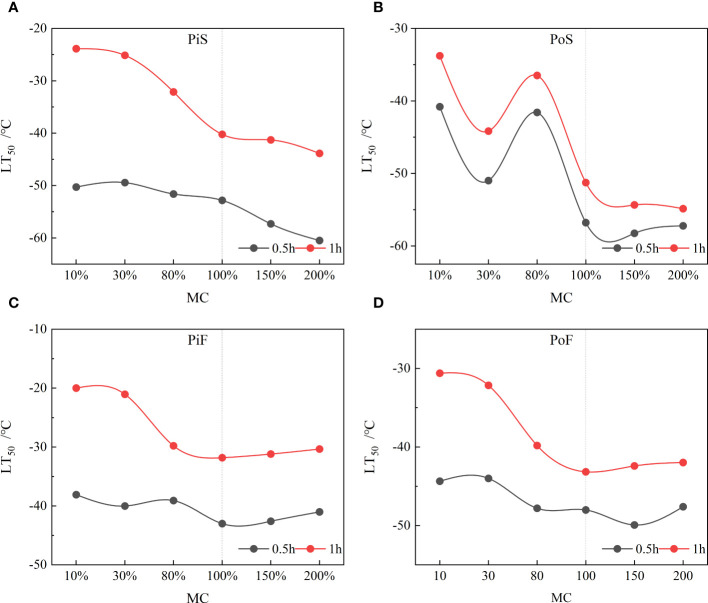
Changing trend of LT50 with MC at all freezing conditions. **(A)** pine–slow cooling rate, **(B)** poplar–slow cooling rate, **(C)** pine–fast cooling rate, and **(D)** poplar–fast cooling rate.

The LT50 values decreased with an increase in the MC to values above the FSP, indicating that the higher water content in the saturated sapwood led to a lower freezing damage temperature (LT50) (**Figure 7**). However, for MC below the FSP or at MC below 30%, no significant changes in LT50 were observed for both cooling rate and freezing duration. The decreasing rate in LT50 of the sapwood tissues was slow when the MC was over 100% for both species. In the slow cooling rate group, the LT50 values of wood frozen for 1 h concentrated around −40°C for pine and around −55°C for poplar, whereas the LT50 values for a freezing duration of 0.5 h concentrated around −60°C for both species. In the fast-cooling rate group, the LT50 values for sapwood tissues with MC over 80% showed approximate agreement for both species, with values LT50 concentrated around −30°C for pine and around −45°C for poplar after 1 h of freezing. For a freezing duration of 0.5 h, the LT50 values stayed around −40°C for pine and around −50°C for poplar. A significant difference of approximately 20°C was observed between the LT50 values of sapwood samples frozen for 1 h (samples with MC levels of 200% and 15%); the LT50 increased considerably when the MC was below 80% in the slow group.

A comparison of the LT50 values between wood exposed to 0.5 h and 1 h of freezing revealed that at an MC above 30%, the LT50 of pine sapwood tissue decreased by over 10°C after 1 h of freezing compared to the case after 0.5 h of freezing at both the cooling rates, whereas the LT50 of poplar sapwood tissue decreased by approximately 5°C after 1 h of freezing compared to the case after 0.5 h of freezing. However, when the MC was below 30%, the difference in LT50 between 0.5 h of freezing and 1 h of freezing was over 20°C for *Pinus* at both the cooling rates, but only about 5°C for poplar at a slow cooling rate. When the MC exceeded the FSP (>80% MC), the LT50 values remained relatively constant at a fast-cooling rate (**Figures 7C, D**), but slightly decreased at a slow cooling rate (**Figures 7A, B**).

**Figure 8:**
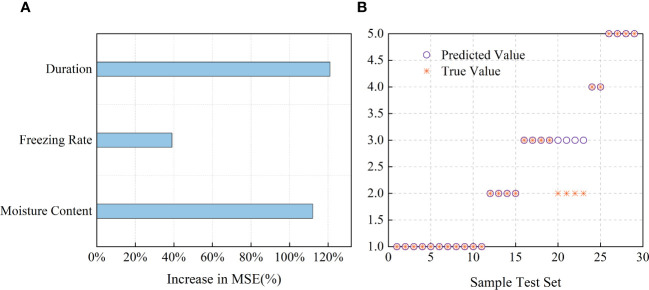
Driven correlations of LT50 with MC, cooling rate and freezing duration based on RF and SVM model simulation. **(A)** Contributions of each factor LT50 based on random forest (RF). Increases in the MSE of variables were used to estimate the importance of each factor, and higher MSE% values imply more important factors. **(B)** Comparison of predicted value and true value for the test set results based on SVM model.

Random forest (RF) and support vector machine (SVM) models were used to model the relationships among the MC, cooling rate, freezing duration, and LT50 values. The coefficients of determination were examined to evaluate the reliability of the models. The analysis results were plotted using Origin2022 (OriginLab, Northampton, MA, USA) (**Figure 8**). The contributions of MC, cooling rate, and freezing duration were tested using the RF and SVM models. The root mean squared error (RMSE) and R^2^ values of the RF model were 0.9945 and 0.8941, respectively (**Figure 8A**). Among the three variables, freezing duration and MC were found to be the dominant factors influencing the LT50 values, whereas the impact of cooling rate was relatively lower. As shown in **Figure 8B**, the penalty coefficient (c) and parameter g of the SVM model were 0.7071 and 45.2548, respectively, and the accuracy of the model was 86.21%.

## 4 Discussion

Whether the intracellular liquid allows cells to recover mainly depends on the speed and intensity of the freezing conditions (Ochandio Fernández et al., 2019). If the exposure temperature is sufficiently low and the freezing duration is sufficiently long, larger ice crystals are formed, causing cell wall rupture and cell death with no chance of recovery. Therefore, the objective of this study was to investigate the semi-lethal temperature for two tree species using the EIS method and the factors influencing this temperature, such as exposure temperature, cooling rate, and cooling duration, through a controlled laboratory experiment. The EIS profiles of frozen Korean pine and Simon poplar sapwood were obtained and the electrical parameters (*r*_e_, *r*_i_, and *τ*) were characterized.

As plant tissues have passive electrical properties, the impedance of biological tissues is primarily related to cellular ion content, cell membrane structure, and viscosity (Jócsák et al., 2019). As the water in plant tissues acts as the conductor of electrical currents, any changes in the state and content of water within the tissues directly affect ion migration, membrane permeability, and other factors, resulting in corresponding changes in tissue impedance (Repo et al., 1997; Ando et al., 2014). Therefore, the significant changes observed in impedance properties of sapwood tissues at freezing temperatures, especially in the range of −40 to −80°C, are reasonable (**Figures 5**, **6A-D**).

The rapid increase in *r*_e_ at temperatures around −40°C (**Figure 5**) was primarily caused by the difference in chemical potential between the extracellular ice and intracellular solution. This difference in chemical potential can lead to cellular dehydration and a consistent increase in the volume of ice crystals, which punctures the plasma membrane, resulting in electrolyte leakage (Imaizumi et al., 2015; Ando et al., 2019). In contrast, the second change in *r*_e_ around −70°C is likely the result of extensive rupture of plasma and organelle membranes, resulting in tissue damage and significant cell death.

The cellular structure of plant tissues can be altered due to changes in water content when exposed to freezing conditions (Mahajan and Tuteja, 2005), along with other factors, such as the species and types of cells and the rate of decrease of the environmental temperature.

HTEs usually occur due to extracellular freezing of water in the ectoplasmic space, xylem conduits, and non-living cells (Neuner et al., 2010; Yu et al., 2017; Hincha and Zuther, 2020). In the DSC curves, HTEs were observed in the sapwood tissues of Korean pine and Simon poplar between −7 and −12°C (Figure). Conversely, LTEs generally occur in xylem parenchyma cells (XPCs) and serve as a strategy in many woody plants to avoid freezing damage through deep supercooling (Neuner et al., 2010; Repo et al., 2022). However, LTEs were not detected in the DSC curves obtained in this study. This does not imply that deep supercooling or intraprotoplasmic freezing does not occur in sapwood tissues. XPCs in tree trunk tissues do not freeze simultaneously during freezing but freeze in small clusters at random locations in the xylem, resulting in only a minimal amount of exothermic release (Neuner et al., 2010). As the sapwood samples used in this study had a lower proportion of XPCs for deep supercooling, LTEs could not be detected using the DSC method.

Although freezing damage in trees is commonly observed at a temperature of approximately −40°C (Repo et al., 2022), the appearance of freezing in XPCs is not solely dependent on environmental temperature but is also influenced by factors such as embolism, cavitation, ion contents of xylem sap, vessel size, cell wall rigidity, degree of lignification, and cell maturation (Wu et al., 2019). Freezing damage in plants primarily occurs as a result of injuries to the cell membranes caused by water freezing into ice crystals and cell dehydration (Pearce, 2001). The electrical properties of plant tissues are significantly affected by their water content across most frequency ranges of EIS (Tiitta et al., 2009; Tomppo et al., 2011; Zelinka et al., 2007). In the present study, the impedance characteristics differed considerably between the different MC groups at the same temperature, especially in terms of the extracellular resistance (*r*_e_), where the difference between MCs above the FSP and below the FSP was at least 100-fold (**Figure 5**). This result aligns with those of previous studies that observed differences in electrical impedance between 20% MC and 12% MC in Southern pine (*Pinus* spp.) (Zelinka et al., 2008).

Extracellular resistance (*r*_e_) can be modeled using a logistic sigmoid function to obtain the LT50 value (Equation 5), allowing EIS to be used as a method to estimate cell membrane injuries in wood tissue (Repo et al., 2021). Maintaining the structural and functional stability of cell membranes during freezing is crucial for the resistance of tissues against mechanical and dehydration stresses and for overcoming complex frost damage (Lenz et al., 2013; Markovskaya and Shibaeva, 2017; Takahashi et al., 2018). The LT50 values of sapwood tissues from Korean pine and Simon poplar with MC above the FSP (approximately 30% MC), and particularly, above 80% MC, were generally lower by approximately 5–15°C than the case for the tissues with MC below the FSP (**Figure 7**). These findings suggest that sapwood tissues have a better ability to supercool (deep supercool) or tolerate freezing-induced cytosolic dehydration when the internal MC is relatively higher. In nature, as free water in cells is susceptible to freezing, some less cold-tolerant plants undergo dehydration during cold acclimation to adapt to freezing temperatures (Ameglio et al., 2015; Karlson et al., 2003; Rajashekar and Panda, 2014; Ouyang et al., 2019b). Even when plant cells are severely dehydrated due to freezing, injury to the cell membrane is reduced because water molecules bound to the cell membrane surface remain spatially separated from the inner cell membrane (Takahashi et al., 2018). Numerous studies have shown that plant tissues have evolved structural adaptations to accommodate the formation of large ice crystals in specific locations within the tissue (Wisniewski et al., 2009). This implies that despite the high-water contents within the tissue, the freezing of water always occurs at specific locations, and the formation of large ice crystals does not harm the tissues for a certain period of time (Wisniewski et al., 2014b). Guy (2003) proposed the control of MC to prevent intracellular freezing; however, this approach is complex and depends on factors such as the amount of liquid state water in the cell, cooling rate, volume ratio, and membrane permeability (Guy, 2003).

In nature, different types of plants typically freeze at relatively slow rates (−1 to 3°C/h) (Arora, 2018). In contrast, freezing of intracellular water under natural episodic frosts is uncommon, but can occur under artificial conditions that involve rapid freezing. For the *Pinus* samples evaluated in this study, the LT50 values were generally higher, ranging from 15 to 20°C, at a fast-cooling rate of 10°C/min than at a slow cooling rate of 2°C/min. The cooling rate had a significant effect on the LT50 values with *p*< 0.01 for both species (**Table 3**). This result indicates that freezing damage occurs earlier when the environmental temperature drops at a faster rate.

Sapwood tissues typically employ two contrasting strategies to cope with freezing stress. When the cooling rate is slow, ice crystals first appear in the extracellular solution, creating a chemical potential difference between the osmotically active water within the cell and the extracellular ice. In contrast, when at fast cooling rates, the sapwood tissue typically resists freezing stresses through supercooling and deep supercooling, which helps prevent the formation of ice crystals (Levitt, 1980). However, at excessively low temperatures, the supercooled metastable liquid can freeze resulting in intracellular freezing that damages the membrane systems and inevitably leads to cell death. In the present study, supercooling and deep supercooling may not have occurred in the sapwood tissues of Korean pine and Simon poplar, as LTEs were not detected (Gusta et al., 1983). This suggests that the chemical potential imbalance induced by freezing stress was responsible for the different LT50 values observed in the two groups subjected to different cooling rates. Furthermore, the rate of cooling that leads to intracellular freezing is influenced by various factors, including the physiological state of the tissue, amount of frozen water in the cell, and permeability of the membrane to water (Arora, 2018). These results further highlight that freezing in cells may be attributed to the interplay of external freezing stress, tissue MC and tissue structure. From the findings of this study, it is evident that even at relatively fast cooling rates of 2°C/min and 10°C/min, some sapwood tissues of Korean pine and Simon poplar were able to survive at temperatures below −40°C. The differences in freezing tolerance exhibited by these tissues is reflected in the LT50 values, as shown in **Table 2**. Weiser (1970) found that at cooling rates of 8 to 10°C/min, leaves already experienced frost damage at −10°C. This suggests that even at very high cooling rates, there may still be subtle differences in the freezing tolerance mechanisms of different plant tissues.

Different durations of freezing can result in varying levels of structural damage. When freezing persists for an extended period, tissue death can occur even if the temperature is considered “non-injurious” (Gusta et al., 1997). The LT50 values were significantly influenced by the duration of freezing (**Table 3**). Generally, for a 1 h freezing duration, the LT50 values were higher by approximately 10–15°C with an MC over 80% and 10–20°C with an MC below 80%, compared to a freezing duration of 0.5 h. These results suggest that tree tissues can tolerate short-versus extreme dehydration during freezing, but not prolonged dehydration (Gusta et al., 1997). Longer durations of freezing lead to the premature death of sapwood tissues, indicating the existence of different freezing tolerance mechanisms for short-versus and long-duration freezes. Min et al. (2014) observed a progressive increase in injury with longer freezing durations in spinach leaves. The findings of this study support the conclusions of a study by Min et al. (2014) and imply that cell injury is not solely influenced by the duration of freezing but is also related to the amount of water exuded during freezing.

Statistical analysis revealed significant interactions between freezing duration and cooling rate (**Table 3**), confirming that cell injury and death are influenced by the interplay of cooling rate and duration (Arora et al., 1992; Waalen et al., 2011). Although extracellular freezing is widely considered the main cause of injury due to cell dehydration, fatal damage resulting from freezing stress and prolonged freezing cannot solely be attributed to dehydration stress. This is likely due to a synergistic effect between different lesions. For example, an increased duration of mechanical stress and strain caused by extracellular ice and cell collapse, leading to shearing at the adhesion sites between the cell membrane and cell wall, can amplify the injurious effects of cellular dehydration over time (Min et al., 2014). Furthermore, the results of the present study suggest that the effects of short-duration and long-duration freezing vary across tree species. The occurrence of deep supercooling and extracellular freezing in the xylem is primarily influenced by the ring porous structure of the tissue and the cell wall thickness, with more elastic cell walls being susceptible to dehydration reactions during freezing. The ability of cells to resist collapse during freezing in response to dehydration is also related to the cell wall thickness (Gusta et al., 1983; Gusta et al., 1997).

Theoretically, the freezing of water in plant tissues cannot occur below −40°C except for the case of intracellular freezing, as the homogeneous freezing temperature of pure water is approximately −38.5°C (Pearce, 2001; Wisniewski et al., 2014a). However, non-homogeneous ice nucleation always occurs in plant tissues due to the presence of ice nucleating active substances in the extracellular space (Wisniewski et al., 2014b). This means that in nature, frost damage occurs earlier in plant tissues. Previous studies have also shown that the LT50 values in stem and seedling tissues of most tree species are concentrated around −40°C (Repo et al., 2022; Wu et al., 2019; Yu et al., 2017). In contrast, in the present study, the extreme LT50 values of sapwood tissues with an MC over 200% for Korean pine and Simon poplar, at slow cooling rates, were concentrated around −60°C (**Figures 7A, B**). This result may be attributed to the presence of dissolved electrolytes in the cells and structural differences between sapwood tissues and other plant tissues, as plant tissues with phloem, wood parenchyma, and meristematic tissue typically have a relatively low LT50 value when subjected to freezing (Lenz et al., 2013).

However, it is noteworthy that −60°C is very close to the extreme temperature for deep supercooling in plant tissues. Deep supercooling in plants can occur anywhere between −15 and −60°C, and −60°C is usually close to the extreme temperature in xylem tissues (Quamme, 1991; Peter, 2012, Wisniewski, 1995; Wisniewski et al., 2014b). In this study, LTEs were not detected during freezing, likely due to prolonged freezing within a certain temperature range, causing all the freezable water within the cells to escape from the xylem cells to distal sites. In contrast, LTEs were detected upon thawing in other species such as red ash (*Fraxinus pennsylvanica* Marsh.) (Gusta, 1983). However, determining the nucleation temperature and supercooling point is not a straightforward task due to its stochastic nature, and estimating these parameters requires 200–300 measurements on individual samples. This is not practical when studying whole plants, as continuous freezing and thawing alter tissue properties (Peter, 2012).

Therefore, the occurrence of freezing damage to plant tissues is influenced by various factors. such as the physiological state and structure of the tissues, MC, carbohydrate composition, cooling rate, and duration of freezing, among others. However, these factors are complex and often do not follow the assumption of a normal distribution and are challenging to effectively correlate with the degree of frost damage. From the relationships established between freezing factors and LT50 of sapwood tissue, it is evident that the MC and freezing duration played a more significant role in determining the change in the LT50 value of wood tissue, whereas the cooling rate had a lesser impact. Although the cooling rates of 2°C/min and 10°C/min are relatively rapid, their effect on the LT50 was different but minimal. However, it is important to consider the interactive effects of the MC, cooling rate, and freezing duration on the temperature at which freezing damage occurs. The R^2^ of the RF model and the accuracy of the SVM model suggest that estimating the occurrence of freezing-induced damage to sapwood tissue based on the MC, cooling rate, and freezing duration is feasible.

## 5 Conclusions

Through the controlled laboratory experiment, the mechanism of freezing injury occurring in trees was investigated. We found that the freezing duration at subzero temperature had the greatest impact on the LT50 of wood tissue, followed by the MC, while the cooling rate had a minimal impact on LT50. Slow cooling combined with long-duration freezing at subzero temperatures led to earlier and more severe freezing damage. The findings of this study could aid the establishment of measures for preventing damage and protecting trees and serve as important references for developing non-destructive and reliable electrical impedance-based methods for detecting freezing damage in trees.

## Data availability statement

The raw data supporting the conclusions of this article will be made available by the authors, without undue reservation.

## Author contributions

XS: Conceptualization, Data curation, Formal Analysis, Writing – original draft, Writing – review & editing. TG: Conceptualization, Methodology, Software, Writing – review & editing. MA: Data curation, Methodology, Resources, Software, Writing – review & editing. SG: Funding acquisition, Supervision, Validation, Writing – original draft, Writing – review & editing.
